# Evaluation of *RAS* Mutational Status in Liquid Biopsy to Monitor Disease Progression in Metastatic Colorectal Cancer Patients

**DOI:** 10.3390/cells12111458

**Published:** 2023-05-24

**Authors:** Elena Lastraioli, Alessandra Bettiol, Jessica Iorio, Elvira Limatola, Daniele Checcacci, Erica Parisi, Cristina Bianchi, Annarosa Arcangeli, Mauro Iannopollo, Francesco Di Costanzo, Marco Di Lieto

**Affiliations:** 1 Department of Experimental and Clinical Medicine, University of Florence, 50134 Florence, Italy; 2 Complex Dynamics Study Centre (CSDC), University of Florence, 50100 Florence, Italy; 3 Medical Oncology, S. Jacopo Hospital, 51100 Pistoia, Italy; 4 Medical Oncology, S.S. Cosma e Damiano Hospital, 51017 Pescia, Italy; 5 Medical Oncology Unit, Azienda Ospedaliero-Universitaria Careggi, Largo Brambilla 3, 50134 Florence, Italy

**Keywords:** *KRAS*, *NRAS*, liquid biopsy, metastatic colorectal cancer

## Abstract

In this study we evaluated both~ K- and N-*RAS* mutations in plasma samples from patients with metastatic colorectal cancer by means of the BEAMing technology, and we assessed their diagnostic performance compared to *RAS* analyses performed on tissue. The sensitivity of BEAMing in identifying *KRAS* mutations was of 89.5%, with a fair specificity. The agreement with tissue analysis was moderate. The sensitivity for *NRAS* was high with a good specificity, and the agreement between tissue analysis and BEAMing was fair. Interestingly, significantly higher mutant allele fraction (MAF) levels were detected in patients with G2 tumors, liver metastases, and in those who did not receive surgery. *NRAS* MAF level was significantly higher in patients with mucinous adenocarcinoma and for those with lung metastases. A sharp increase in the MAF values was observed in patients who moved towards disease progression. More strikingly, molecular progression always anticipated the radiological one in these patients. These observations pave the way to the possibility of using liquid biopsy to monitor patients during treatment, and to enable oncologists to anticipate interventions compared to radiological analyses. This will allow time to be saved and ensure a better management of metastatic patients in the near future.

## 1. Introduction

ColoRectal Cancer (CRC) represents a major public health issue, being the third most frequent malignant tumor in both sexes, accounting for 10% of the cases worldwide and the fourth leading cause of cancer death, causing 9.2% of deceases worldwide [1,2]. The gold standard of treatment for CRC patients is represented by surgery but metastatic (TNM IV, mCRC) patients are also treated by systemic approaches, based on chemotherapy, targeted therapy, and combination therapies, although frequently characterized by reduced effectiveness [3]. For this reason, to achieve treatment optimization, different biomarkers have been proposed [3]. According to the National Comprehensive Cancer Network (NCCN) guidelines updated in 2022, therapeutic selection must take into account molecular features, including *RAS*, *EGFR*, and *BRAF* mutations, MSI, CpG island methylation; P21, SCNA, PTEN, and TS expression [4]. In the clinical practice, *KRAS* and *EGFR* mutations are considered the most relevant although they are present roughly in 40 and 3% of mCRC, respectively [5].

It has been clearly shown that the occurrence of *RAS* and *BRAF* mutations are the main elements responsible of the failure of anti-EGFR-based therapy, such as cetuximab and panitumumab [6,7]. For this reason, before defining a therapy schedule for mCRC patients, the presence of *BRAF*, *KRAS*, and *NRAS* mutations is routinely investigated in tissue biopsies in order to select the patients most likely to respond to anti-EGFR therapy [8,9,10]. Typically, the evaluation of *RAS* and *BRAF* mutational status requires the acquisition of tumor tissue, the subsequent processing to formalin-fixed paraffin-embedded (FFPE) specimens, and molecular testing with various techniques, with consequent limitations in studying a single snapshot of a tumor due to both tumor heterogeneity and treatment associated evolution. Therefore, a single biopsy is likely to underestimate the complexity of the tumor genomic landscape [11]. These issues might be overcome by analyzing circulating tumor DNA (ctDNA) representing a variable and small fraction of the total circulating cell-free DNA (cfDNA) that can be found in the plasma of the patients [12,13]. Notably, the detection of a low amount of mutated ctDNA through the implementation of ultrasensitive assays in clinical routine could reduce the need for second biopsies and anticipate radiological progression.

ctDNA levels are associated with biological and clinicopathological features such as tumor burden, stage, histotype, apoptotic rate, blood vessel proximity, and metastatic potential [14,15,16]. A high proportion of mCRC patients are characterized by measurable ctDNA in plasma and 1.9–27% harbor mutations [15]. Hence, the non-invasive detection of emerging *KRAS* mutations in cfDNA from peripheral blood can help to detect resistance to anti-EGFR therapy [17]. Specifically, high levels of *KRAS* mutant allele fraction (MAF) might be associated with a poor outcome for patients treated with cetuximab [18,19]. A fraction of patients without *KRAS* and *NRAS* mutations treated with anti-EGFR might develop *RAS* mutations as soon as the disease progresses [17,20,21,22,23,24,25,26,27]. More importantly, the occurrence of *RAS* mutations in cfDNA can be detected before clinical progression of the disease [17], and, thus, anti-EGFR treatment should be stopped when *RAS* mutations are detected and a rechallenge could be carried out when the mutational status becomes wild type again [26].

The aims of the present paper were the following: (a) evaluate the concordance between *KRAS* and *NRAS* mutational status in tissue and plasma in a cohort of mCRC patients, and test the diagnostic performance of plasma as compared to tissue analyses; (b) evaluate the mutant allele fraction (MAF) distribution in plasma samples and search for possible clinical correlations; and (c) monitor *RAS* mutational status at different time points during treatment until disease progression.

## 2. Materials and Methods

### 2.1. Study Design, Population, and Setting

The present study is a biological, observational, prospective, multi-center, open-label translational study involving the collection of blood samples and clinical data from mCRC patients treated for metastatic disease. The study was conducted among patients enrolled at the Units of Medical Oncology of the Careggi University Hospital (Florence, Italy), Medical Oncology of the S. Jacopo Hospital (Pistoia, Italy), and Medical Oncology of the S.S. Cosma e Damiano Hospital (Pescia, Italy) between March 2017 and August 2022.

Patients were considered eligible if they had a histological diagnosis of colorectal adenocarcinoma stage IV TNM, were treatment naïve, and had measurable disease (according to Response Evaluation Criteria in Solid Tumours (RECIST) criteria v.1.1) [28].

The study was approved by the local ethical committee (BIO.16.028 released on 5 October 2016 for Careggi hospital and 15858_bio, released on 5 March 2020 for Pistoia and Pescia hospitals); each patient provided informed written consent at the enrollment. 

### 2.2. Patients’ Assessment and Follow-Up

Demographic, clinical, and therapeutic features of the patients were retrieved from the medical charts at time of inclusion in the study. 

For all patients, data on tissue *KRAS* and *NRAS* status were retrieved; indeed, for all the patients, *KRAS* and *NRAS* status had been previously determined in FFPE tumor tissue biopsies of either primary tumors or metastases by next generation sequencing (NGS), conducted by experienced personnel at the abovementioned hospitals as a routine procedure. According to clinical practice, patients with wild type (WT) *RAS* were treated with anti-EGFR +/− chemotherapy on physician’s choice. mCRC patients with mutated *RAS* on tissue analyses were treated with anti-VEGF biologics +/− synthetic chemotherapy, depending on the physician’s choice. First-line treatment was given until disease progression or unacceptable toxicity.

Computed tomography (CT) radiological evaluation was performed before starting first-line (baseline) treatment and every 3 months until progression, according to clinical practice, to monitor response. Data on all-cause mortality were also prospectively recorded. 

### 2.3. Sample Collection

**Figure 1:**
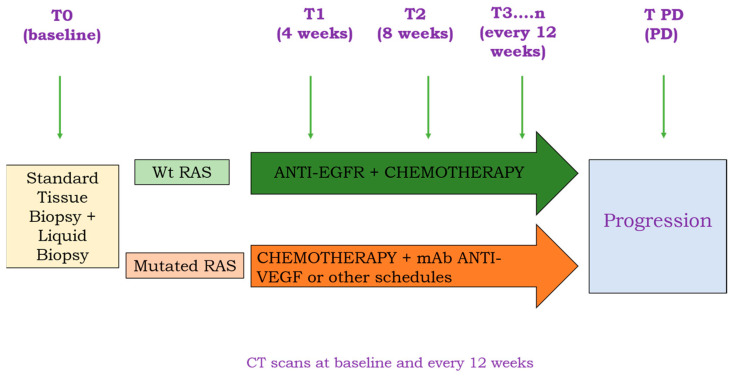
Schematic representation of the study design. T (time of blood collection): 0 (baseline, at the enrollment); 1 (at 4 weeks after treatment start); 2 (after 8 weeks of treatment); PD (at disease progression).

Blood samples for ctDNA analysis were collected prior to starting first-line treatment (T0), 4 (T1), 8 weeks (T2) after starting treatment, and every 12 weeks thereafter (T3……n) until disease progression (T PD), as shown in Figure 1. A plasma sample was also collected at the time of radiological progression according to RECIST version 1.1 criteria [28].

For each patient enrolled in the study, 8 mL of peripheral blood was collected in either K_2_ EDTA BD Vacutainer^®^ collection tubes (BD, Franklin Lakes, NJ, USA) or Cell Free DNA BCT collection tubes (Streck, La Vista, NE, USA) by the nurses of Medical Oncology units of the abovementioned hospitals, and this was taken immediately before starting therapy. Plasma was then prepared within 4 or 72 h, depending on the collection tubes used, and according to the protocol released by Sysmex-Inostics for the determination of *KRAS* and *NRAS* status with OncoBEAM^®^ RAS CRC assay (Sysmex Inostics, Hamburg, Germany). Plasma samples were stored at −80 °C. 

### 2.4. ctDNA Extraction and Purification

ctDNA was extracted and purified using Qiagen’s QIAamp^®^ circulating nucleic acid kit and QIAvac24 plus (Qiagen, Hilden, Germany) with modifications to the manufacturer’s protocol, as indicated by Sysmex Inostics.

### 2.5. BEAMing

For the detection of *RAS* mutations in ctDNA, the OncoBEAM^®^ RAS CRC kit (Sysmex Inostics, Hamburg, Germany) was used, following the supplier’s protocol. OncoBEAM^®^ RAS CRC kit (Sysmex Inostics, Hamburg, Germany) is able detecting 34 mutations in different codons of *KRAS* and *NRAS*. ctDNA extracted from plasma samples were amplified through a multiplex PCR, and samples then pooled and properly diluted were amplified through emulsion PCR. After the completion of the emulsion PCR, the drops were broken and the amplicons were retrieved, since they are bound to the magnetic beads. Subsequently, samples were hybridized with specific fluorescent probes and the fluorescent signals were then detected by Cube16 flow cytometer. Finally, data were analyzed by FCS Express software version 5.0. 

### 2.6. Statistical Analysis 

Categorial variables were reported as absolute frequencies and percentages, and continuous variables as median value and interquartile range (IQR). The Shapiro–Wilk test was used to test the normality assumption for data distribution. 

The diagnostic performance of plasma BEAMing was assessed, considering tissue *KRAS* and *NRAS* analyses as a reference standard; sensitivity, specificity, positive predicted value (PPV), negative predictive value (NPV), and related 95% confidence intervals (CI) were estimated. The level of agreement between plasma and tissue *KRAS* and *NRAS* analyses was also evaluated using Cohen’s k test and its 95% CI.

Differences in the therapeutic response or survival in patients with WT or mutated plasma and tissue *KRAS* and *NRAS* were assessed using the Fisher exact test for unpaired data. Differences in median MAF *KRAS* and *NRAS* levels according to demographic, clinical, therapeutic, and outcome data were assessed, and compared using the Mann–Whitney test or the Kruskal–Wallis test for unpaired data, as appropriate. 

In a post-analysis analysis, Receiving Operating Characteristics (ROC) curves were derived to assess the Area Under the ROC Curve (AUC) of MAF *KRAS* and *NRAS* levels in discriminating CRC patients with liver and lung metastases, respectively. Empirical estimation of the optimal cut-point for MAF *KRAS* and *NRAS* as a possible diagnostic test was computed using the Youden method. 

Statistical significance was considered for *p*-values < 0.05. All analyses were conducted using the software Stata (StataCorp, version 14).

## 3. Results

Sixty-two patients suffering mCRC were enrolled; of them, 35 were men (56.5%), with a median age at inclusion of 67 (61–74) years. The demographic, clinical, and therapeutic features of the patients are summarized in Table 1.

### 3.1. KRAS and NRAS Mutational Status in Tissue Samples

**Figure 2:**
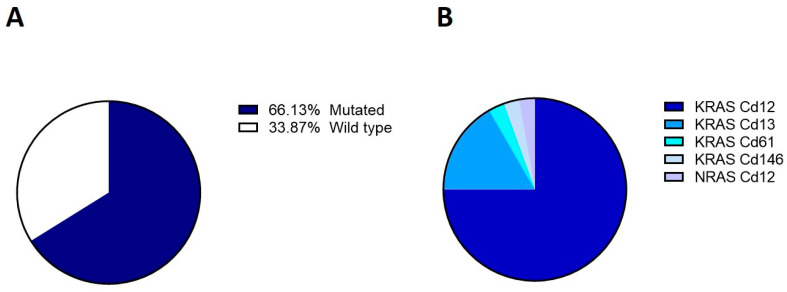
*K*-and *NRAS* profile in tumor tissue. (**A**) Frequency of *RAS* genotype in the cohort under study; (**B**) Frequency of *K*-and *NRAS* mutations.

At molecular analysis of *KRAS* and *NRAS* in FFPE tumor tissue biopsies, 41 out of 62 patients (66.1%) harbored *RAS* mutations, while 21 (33.9%) were classified as WT. Only one patient (1.6%) showed *NRAS* mutation in the tissue (Figure 2). Information on *BRAF* mutational status in tissue biopsies was also available for 51/62 patients. Most patients displayed a WT *BRAF* (n = 46/51; 90.2%), with 5/51 (9.8%) presenting a mutated *BRAF*; all five patients with mutated *BRAF* were WT for *KRAS* and *NRAS* in tissue biopsies.

### 3.2. KRAS and NRAS Mutational Status Evaluation by BEAMing

*RAS* mutational status at the baseline were evaluated through BEAMing for all the patients whose plasma samples had appropriate quality and quantity (56 for *KRAS*; 61 for *NRAS*).

**Figure 3:**
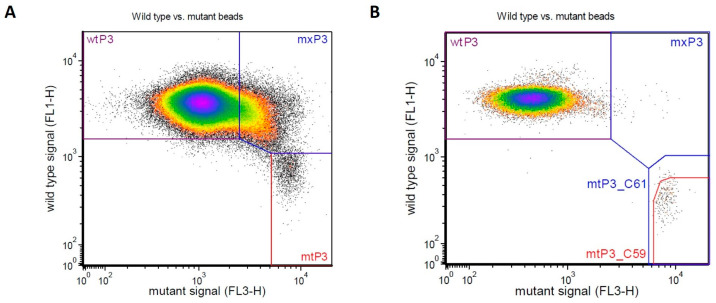
*K*-and *NRAS* mutations detected by OncoBEAM^®^ RAS CRC assay in representative samples. (**A**) *KRAS* Codon 12; (**B**) *NRAS* Codon 61 (gated along with Codon 59, as per manufacturer’s specifications).

Overall, 43 out of 56 plasma samples were found to harbor *KRAS* mutations. As expected, *KRAS* codon 12 was confirmed to be the most frequently affected site in the cohort of patients under study, since mutations at this level were present in 38 out of 43 the mutated baseline samples. As for *NRAS*, only 6 out of 61 samples were found to harbor mutation at codon 12 (3 samples) and codon 61 (3 samples). Representative plots of samples harboring *KRAS* (codon 12) and *NRAS* (codon 61) mutations detected by BEAMing are shown in Figure 3.

In the dot plots obtained through flow cytometry, as for those reported in Figure 3, the mutant beads are present in the bottom right gate at variable extent, depending on the MAF values. The evaluation of the same samples was also carried out by a different technique and similar results were obtained (Lastraioli E et al., manuscript in preparation). 

#### Diagnostic Performance and Concordance between Tissue and Plasma KRAS and NRAS

As a preliminary step, the concordance and diagnostic performance of *KRAS* and *NRAS* analysis as compared to tissue analyses was evaluated (Table 2a,b; Table 3a,b). 

The sensitivity of BEAMing in identifying mutated *KRAS* was of 89.5% (95% CI 75.2–97.1%), with a fair specificity [50.0% (26.0–74.0%)], and PPV and NPV of 79.1% (70.2–85.9%) and 69.2% (44.4–86.4%), respectively.

Coherently, the agreement between tissue analysis and BEAMing was moderate [76.8%, Cohen’s k: 0.43 (0.17–0.68)] (Table 2a), with a similar concordance for the identification of the different codons [75.0%, Cohen’s k: 0.54 (0.33–0.75)] (Table 2b). 

As for *NRAS*, the sensitivity of BEAMing in identifying mutated *NRAS* was high [100% (2.5–100%)], with a good specificity [91.7% (81.6–97.2%)] and NPV of 100%, but with a low PPV [16.7% (8.0–31.6%)]. The agreement between tissue analysis and BEAMing in identifying WT or mutated *NRAS* was fair (91.8% Cohen’s k: 0.27 (−0.15–0.68)) (Table 3a), with a similar concordance for the identification of the different codons (93.4%, Cohen’s k: 0.32, 0.15–0.80) (Table 3b). 

Notably, the proportion of patients with concordant plasma and tissue *KRAS* or *NRAS* did not significantly differ according to sex, histology, grading, site of primary tumor, staging, site of metastasis (liver, peritoneum, lung, lymph nodes, locoregional), number of sites with metastasis, surgery, type of chemotherapy, or outcome or survival.

### 3.3. KRAS and NRAS Status and Clinical Outcomes

We further investigated whether *KRAS* or *NRAS* status was associated with clinical outcomes, including response to treatments and mortality. Information on the response to treatments was available for 43 out of 62 patients. Overall, three patients achieved complete response (7.0%), four partial response (9.3%), and eight maintained a stable disease (18.6%). Conversely, cancer progression was reported in 20 (46.5%), while in eight patients, the evaluation was not performed since it was too early (TE, 18.6%). Survival data were available for 36 out of 62 patients. After a median of 254 days (IQR 95–447) following inclusion in this study, 25 patients were still alive (69.4%) while 11 died (30.6%). 

No significant difference in treatment outcome or survival was reported between patients with WT or mutated *KRAS* or *NRAS* (Supplementary Tables S1a,b and S2a,b). 

As discussed in the introduction to this manuscript, RAS mutational status drives the choice of chemotherapic agents, particularly anti-EGFR, in clinical practice, as RAS mutation is associated with a poor response to anti-EGFR therapies. We therefore assessed the clinical response to anti-EGFR therapies in patients with WT RAS at tissue analyses but with mutated RAS at BEAMing (nine for KRAS and five for NRAS, including two patients with both KRAS and NRAS discordance). Disease progression was reported in four out of nine patients (44.4%) with discordant KRAS, and three out of four patients (75%) with discordant NRAS (for the fifth patient, data on response to treatment was not available). Three patients with discordant KRAS/NRAS received anti-EGFR treatment, and one of them experienced a disease progression. Notably, this patient had WT NRAS at tissue analysis but mutated NRAS at plasma analyses, with a MAF level of 0.516 and codon 61 mutation. 

### 3.4. Assessment of KRAS and NRAS Mutant Allele Fraction

We further quantified the MAF (Table 4). The median MAF level for *KRAS* was of 0.16 (IQR 0.01–4.79; range 0–28.15). Notably, significantly higher levels were detected in patients with G2 tumor grading [0.49 (0.02–7.37)] as compared to those with G3 [0.01 (0.01–0.14)] or G4 (0.00) (*p* = 0.025). Higher MAF levels were also found in patients with liver metastasis [0.33 (0.02–6.76), as compared to 0.05 (0.01–0.44) in those without; *p* = 0.049], and in those who did not undergo surgery at site of primary tumor [5.46 (0.07–9.86), as compared to 0.06 (0.01–0.92) in those who underwent surgery; *p* = 0.010]. 

Regarding *NRAS*, the median level in the overall cohort was of 0.007 (IQ1 0.003–0.010; range 0.001–0.516). This level was significantly higher for patients with mucinous adenocarcinoma [0.027 (0.009–0.310), as compared to 0.006 (0.002–0.008) for those with adenocarcinoma; *p* = 0.004] and for those with lung metastasis [0.008 (0.006–0.017), as compared to 0.005 (0.002–0.009) for those without; *p* = 0.025]. 

We speculated that *KRAS* and *NRAS* levels of MAF in plasma might be a biomarker to early detect liver and lung metastases in CRC patients, respectively. Thus, a post hoc analysis was conducted to investigate the performance of MAF *KRAS* in identifying patients with liver metastasis, but the AUC was poor (0.66, 95% CI: 0.51, 0.80). An empirical cut-off of MAF *KRAS* of 0.196 was found to be optimal, but displayed a poor sensitivity (0.58) and specificity (0.68). Similarly, we assessed the performance of MAF *NRAS* in identifying patients with lung metastasis. An AUC of 0.68 (95%CI: 0.54–0.82) was found, the optimal empirical cut-off of MAF *NRAS* being 0.006, with a moderate sensitivity (0.78) and a poor specificity (0.51).

### 3.5. Monitoring of KRAS and NRAS Mutational Status over Time

For a subset of patients (n = 31), the MAF status was re-evaluated every 4 weeks from the beginning of the therapy (at 4 weeks, 8 weeks, and 12 weeks, and until the eventual progression of the disease). In the majority of patients, the presence or absence of mutations in *KRAS* and *NRAS* was maintained during the course of therapy. However, in some cases, variations are observed. The MAF values are reported in Table 5. 

**Figure 4:**
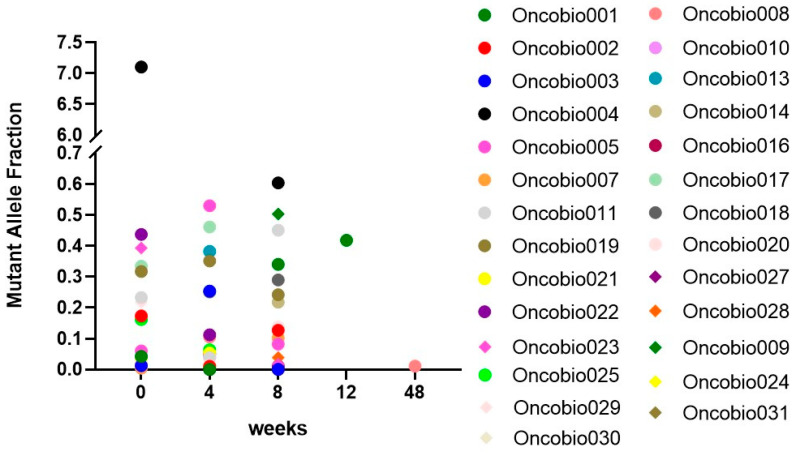
Scatter plot showing the distribution of the Mutant Allele Fraction values during the therapy (at the baseline and after 4, 8, and 12 weeks).

The values of MAF were plotted as a scatter plot for all the patients analyzed at the different follow-up timepoints (Figure 4). As can be observed, there is a wide variability between the samples, although the great majority of them fall into the 0.0–0.5 range. 

**Figure 5:**
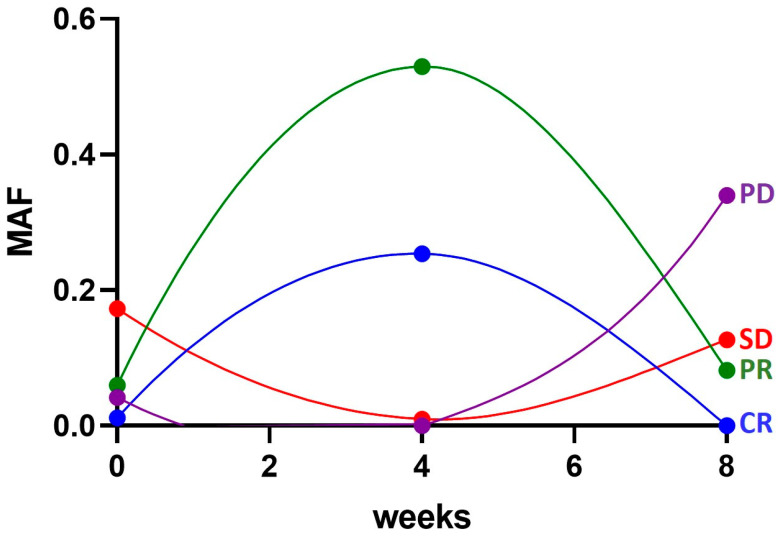
Akima spiline plots showing the distribution of the Mutant Allele Fraction values over time (at the baseline and after 4 and 8 weeks of treatment) for four representative patients with different best response. PD: Progressed Disease; SD: Stable Disease; PR: Partial Response; CR: Complete Response.

For some of the patients, at least three evaluations were available, and, thus, MAF values were plotted in the graphs shown in Figure 5, reporting the number of weeks of treatment on the *x* axis and MAF values on the *y* axis.

The curves shown in Figure 5 represent four different possible responses to therapy that turned out to be associated with MAF trend. As can be noticed, the patient who received a complete response (blue curve) had quite low MAF levels at the baseline with a sharp increase at four weeks (that might be due to the efficacy of the therapy to eliminate wild type clones) followed by a decrease to zero at eight weeks. Similarly, the patient who got a partial response (green curve) had a similar trend but the MAF levels did not reach zero. The red curve is representative of a patient who had stable disease and in this case the baseline and 8-week MAF were comparable. Finally, the fourth case is that of a patient whose disease progressed (purple curve): the baseline MAF was low and with the treatment and it fell to zero, but after four weeks it started increasing rapidly and sharply. 

**Figure 6:**
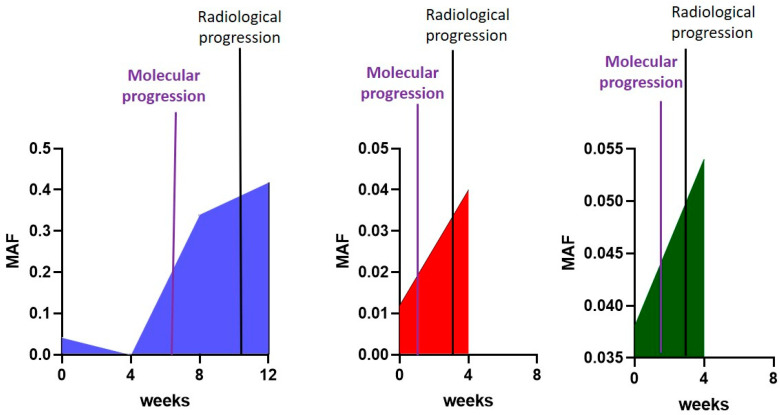
Mutant Allele Fraction values over time (at the baseline and after 4, 8, and 12 weeks of treatment) for three representative patients with Progressed Disease.

Based on these observations, we then focused on patients whose disease was progressed to increased malignancy. In Figure 6, graphs of three representative patients are reported: for all of them, a sharp increase in the MAF values can be observed, confirming what was described for the purple curve in Figure 5. Additionally, when the dates of radiological and molecular progression were taken into account, it emerged that molecular progression (purple lines) always anticipated the radiological one (black lines).

Another interesting finding is represented by the detection of a double mutation in four samples (namely, Oncobio001 at 12 weeks, Oncobio017 at 4 weeks, Oncobio021 at baseline, and Oncobio030 at 4 weeks) (see Table 4). For Oncobio001 and 17, both mutations were detected in *KRAS* (codons 12 + 61 and codons 12 + 117, respectively), while in Oncobio021 and Oncobio030, one mutation was detected in *KRAS* (codon 12) and the other was in *NRAS* (codon 12).

## 4. Discussion

This study evaluated *RAS* mutations in plasma samples from patients with mCRC by the means of BEAMing technology, and assessed its diagnostic performance as compared to tissue analyses on tumor biopsies. The clinical value of monitoring plasma *RAS* mutational status during treatment was also investigated.

Assessing *K*- and *NRAS* mutational status in tumor biopsies is a common procedure in clinical practice, with relevant implications in the choice of the most appropriate pharmacological approach [8,9,10]. Indeed, mutations in these genes have been associated with a poor response to anti-EGFR therapies, and the assessment of *K-* and *NRAS* mutational status can therefore help in maximizing the likelihood of a patient’s response to chemotherapy [6,7]. In our cohort, the molecular evaluation of *RAS* mutational status in tumor tissue samples, performed by NGS, confirmed a low frequency of both *KRAS* [29] and *NRAS* mutations in mCRC patients, which is in agreement with the data reported in the literature for this type of tumor (1–5% for mCRC) [30].

The assessment of the allelic configuration of mutant oncogenes and their MAF in oncologic patients is relevant since mutations in driver oncogenes could influence drug response and resistance. This is, for instance, the case with *KRAS* [31,32]. In vivo data indicate that *KRAS*-mutant tumors have increased in proliferation and sensitivity to MEK inhibitors with respect to wild type tumors [32,33]. Nevertheless, it should be pointed out that although mutations in driver oncogenes are associated with diverse outcomes, MAF levels have not been shown to have an impact on survival or to help in predicting the response to targeted therapy in metastatic patients [34].

It is known that *K-* and *NRAS* mutational profiles should not only assessed at baseline but also monitored during follow-up in order to anticipate treatment outcomes. However, considering the general health conditions of metastatic patients, it is not bearable to manage it through tissue biopsies. For this reason, in recent years, evidence has been gathered concerning the importance of liquid biopsy as a surrogate of standard tissue biopsies for diagnostic purposes as well as for monitoring mCRC patients. Indeed, liquid biopsy can represent a minimally invasive and valuable tool for monitoring mCRC patients undergoing therapy.

In 2016, a meta-analysis was published showing that ctDNA represents an indicator for poor prognosis (both recurrence free survival, RFS, and overall survival, OS) in CRC patients [35]. In particular, an interesting study performed by Spindler et al. in 2014 [19] demonstrated that cfDNA increase had an impact on both PFS and OS. Moreover, by performing a parallel analysis of ctDNA and Circulating Tumor Cells (CTCs), it was shown that the former represents a better tool for CRC patients’ management, since ctDNA, but not CTCs, were detected in all the samples and a low volume of blood was sufficient for molecular analysis [36].

However, the concordance and diagnostic performance of BEAMing as compared to traditional tissue analyses is still a matter of debate [13,27,37]. Our results indicate a sensitivity for BEAMing in identifying *KRAS* mutations of 89.5%, with a fair specificity and a moderate agreement with tissue analysis. Conversely, the sensitivity for *NRAS* was high, with a good specificity, although the agreement was fair. It can be speculated that discordant *KRAS* or *NRAS* analyses, particularly in the case of WT tissue and mutated plasma results, can have a relevant clinical implication, as patients who are not candidates for anti-EGFR therapies might be treated with these agents, which are poorly effective in case of *RAS* mutation.

Regarding the double mutations found in some samples, although such a condition is infrequent and generally *K*- and *NRAS* mutations are mutually exclusive, the high sensitivity of BEAMing technology actually made it possible to detect subclonal mutations with extremely low frequency. It is worth noting that two of the three patients in which a double mutation was detected went towards disease progression, as already published by our group for another patient [26].

In addition to mutational status, we also quantified MAF levels in plasma in search of a possible association with clinical features. Significantly higher *KRAS* MAF levels were detected in patients with G2 tumor grading, liver metastasis, and in those who did not undergo surgery at site of primary tumor; as for *NRAS*, significantly higher levels were found in patients with mucinous adenocarcinoma or with lung metastasis. Nevertheless, both *KRAS* and *NRAS* MAF displayed a poor diagnostic performance in identifying patients with liver and lung metastasis, respectively, and their potential role as a diagnostic biomarker for early detection of metastasis in CRC patients is unclear. As MAF levels were quantified only in plasma and not in tissue biopsies, no correlation analysis could be performed between MAF levels in the two samples, either.

Routine monitoring of *RAS* mutational status and MAF levels is gaining importance in clinical practice in order to predict treatment outcomes early. In the majority of the patients analyzed in this study, the presence or absence of mutations in *KRAS* and *NRAS* was maintained during the course of therapy. However, in some cases, variations were observed, and taking into account the MAF values, more information can be derived. In general, a sharp MAF increase was associated with disease progression, in accordance with the published data, which referred to both *RAS* and other genes in CRC [24,38,39] and other tumors [40], such as, for example, pancreatic [41,42], lung [43], and breast cancer [44,45,46,47] detected by BEAMing or other techniques. Our data are in accordance with published results, since in CRC, it was shown that ctDNA levels decreased after surgery but might be detectable after 15–50 days, and the presence of mutations correlated to disease recurrence [48]. Our data represent a confirmation of the pilot work carried out by Misale et al. in a small cohort of CRC patients [49], too, since they reported that *KRAS* mutations could be detected in plasma 10 months before the radiological progression. Our data are obtained in a bigger cohort and with an optimized BEAMing protocol, but the same conclusions are derived from such analysis, as is the paper of Toledo et al. [24].

## 5. Conclusions

Taken together, our findings show that determining the molecular profile of the tumor becomes essential when dealing with mCRC patient treatment. Therefore, the development of a real-time molecular monitoring of tumor characteristics during sequential therapies could be a successful strategy in the direction of molecularly guided precision therapy, allowing clinicians and patients to gain considerable advantages that avoid unnecessary toxic effects and economic costs for ineffective treatment choices [26]. In fact, the possibility of success of a precision medicine approach therapy, choosing a specific molecular target, such as EGFR, and using monoclonal antibodies against it is strictly associated with the maintenance of a wild type status of *RAS* genes.

Moreover, the demonstration that molecular progression precedes the radiological one is particularly relevant, since it opens the possibility to use liquid biopsy to monitor patients during treatment and to give the oncologists the opportunity of a rapid intervention when disease starts progressing.

To this purpose, the molecular analysis of ctDNA from plasma, obtained through liquid biopsy, and performed with OncoBEAM RAS CRC assay, represent a great tool in order to study the mutational profile of biomarkers of responsiveness to targeted therapy, employing a minimally invasive approach, which is particularly important when it comes to treating metastatic patients.

## Figures and Tables

**Table 1:** Demographic, clinical, and molecular features of the patients enrolled in the study.

	Tot (n = 62) *
**Demographics**	
Male sex	35 (56.5%)
Age at inclusion, median (IQR)	67 (61–74)
**Histology**	
Adenocarcinoma	55 (88.7%)
Mucinous adenocarcinoma	7 (11.3%)
**Grading**	
G2	26 (41.9%)
G3	11 (17.7%)
G4	1 (1.6%)
Missing	24 (38.7%)
**Site of primary lesion**	
Colon	46 (74.2%)
Rectal	10 (16.1%)
Transverse colon	2 (3.2%)
Missing	4 (6.5%)
**Staging**	
IV (new diagnosis)	21 (33.9%)
IV (relapse)	14 (22.6%)
Missing	27 (43.6%)
**Number of metastases**	
1	28 (45.2%)
2	24 (38.7%)
3+	10 (16.1%)
**Site of metastasis**	
Liver	37 (59.7%)
Lung	18 (29.0%)
Loco-regional	12 (19.4%)
Lymph nodes	12 (19.4%)
Peritoneum	12 (19.4%)
Pleura	5 (8.1%)
Adrenal gland	3 (4.8%)
Bone	2 (3.2%)
Kidney	2 (3.2%)
Pancreas	1 (1.6%)
Endometrium	1 (1.6%)
Bladder	1 (1.6%)
Brain	1 (1.6%)
**Surgery on primary site**	48 (77.4%)
**Chemotherapy**	
Yes	47 (75.8%)
No	1 (1.6%)
Missing	14 (22.6%)
**Chemotherapy agents**	
Only synthetic agents	17 (27.4%)
Only targeted biologics	5 (8.1%)
Combination of synthetic and biologics	25 (40.3%)

* n (%) or median (IQR).

**Table 2:** (**a**,**b**) Diagnostic performance of *KRAS* detected in plasma as compared to *KRAS* detected in tissue.

(**a**)					
	**Tissue *KRAS***				
	**WT**		**Mutated**		**Value (95% CI)**
**Plasma *KRAS***					
**WT**	9 (14.5%)		4 (6.5%)		Cohen’s K: 0.43 (0.17–0.68)
**Mutated**	9 (14.5%)		34 (54.8%)		Sensitivity: 89.5% (75.2–97.1%)
					Specificity: 50.0% (26.0–74.0%)
**Not Informative**	3 (4.8%)		3 (4.8%)		PPV: 79.1% (70.2–85.9%)
					NPV: 69.2% (44.4–86.4%)
(**b**)					
	**Tissue *KRAS***				
	**Cd12**	**Cd13**	**Cd146**	**WT**	**Concordance**
**Plasma *KRAS***					
**Cd12**	28 (45.2%)	1 (1.6%)	0	9 (14.5%)	Kappa: 0.54 (95% CI: 0.33–0.75); % agreement: 75%
**Cd13**	0	4 (6.5%)	0	0	
**Cd146**	0	0	1 (1.6%)	0	
**WT**	3 (4.8%)	1 (1.6%)	0	9 (14.5%)	
**Not Informative**	3 (4.8%)	0	0	3 (4.8%)	

**Table 3:** (**a**,**b**) Diagnostic performance of plasma *NRAS* as compared to tissue *NRAS*.

(**a**)			
	**Tissue *NRAS***		
	**WT**	**Mutated**	**Value (95% CI)**
**Plasma *NRAS***			
**WT**	55 (88.7%)	0	Cohen’s K: 0.27 (−0.15–0.68)
**Mutated**	5 (8.1%)	1 (1.6%)	Sensitivity: 100.0% (2.5–100%)
			Specificity: 91.7% (81.6–97.2%)
**Not Informative**	1 (1.6%)	0	PPV: 16.7% (8.0–31.6%)
			NPV: 100%
(**b**)			
	**Tissue *NRAS***		
	**Cd12**	**WT**	**Concordance**
**Plasma *NRAS***			
**Cd12**	1 (1.6%)	2 (3.2%)	Kappa: 0.32 (95% CI: −0.15–0.80); % agreement: 93.4%
**Cd61**	0	3 (4.8%)	
**WT**	0	55 (88.7%)	
**Not Informative**	0	1 (1.6%)	

**Table 4:** Mutant allele fraction (MAF) of *KRAS* and *NRAS*, overall and stratified according to the main demographic, clinical, and therapeutic features.

		MAF *KRAS* (Median, IQR)	*p*-Value §		MAF *NRAS* (Median, IQR)	*p*-Value §
**Overall**	**n = 55**	0.16 (IQR 0.01–4.79; range 0–28.15)		n = 61	0.007 (IQ1 0.003–0.010; range 0.001–0.516)	
**Demographics**						
Male sex	n = 23	0.22 (0.01–5.46)	0.511	n = 34	0.006 (0.003–0.009)	0.425
Female sex	n = 32	0.06 (0.01–2.05)		n = 27	0.007 (0.002–0.014)	
**Histology**						
ADK	n = 48	0.17 (0.02–3.90)	0.990	n = 54	0.006 (0.002–0.008)	0.004 *
Colloid ADK	n = 7	0.05 (0.01–13.0)		n = 7	0.027 (0.009–0.310)	
**Grading**						
G2	n = 242	0.49 (0.02–7.37)	0.025 *	n = 25	0.005 (0.003–0.010)	0.828
G3	n = 8	0.01 (0.01–0.14)		n = 11	0.007 (0.002–0.011)	
G4	n = 1	0.00		n = 1	0.006	
Missing	n = 22	0.25 (0.03–3.10)		n = 24	0.007 (0.004–0.009)	
**Site of primary lesion**						
Colon	n = 43	0.22 (0.01–5.46)	0.660	n = 45	0.007 (0.003–0.010)	0.189
Rectal	n = 8	0.06 (0.04–0.60)		n = 10	0.007 (0.003–0.010)	
Transverse colon	n = 2	0.04 (0.01–0.06)		n = 2	0.002	
Missing	n = 2	1.62 (1.62–3.10)		n = 4	0.007 (0.005–0.007)	
**Staging**						
IV (new diagnosis)	n = 20	0.71 (0.04–9.01)	0.330	n = 20	0.008 (0.006–0.105)	0.575
IV (relapse)	n = 12	0.48 (0.01–5.07)		n = 14	0.011 (0.004–0.030)	
Missing	n = 23	0.06 (0.01–0.33)		n = 27	0.004 (0.002–0.007)	
**Number of metastases**						
1	n = 26	0.23 (0.01–1.00)	0.776	n = 28	0.005 (0.002–0.008)	0.243
2	n = 21	0.06 (0.01–7.66)		n = 23	0.007 (0.003–0.010)	
3+	n = 8	0.15 (0.04–5.07)		n = 10	0.007 (0.004–0.014)	
**Site of metastasis**						
Liver	No: n = 22; Yes: n = 33	No: 0.05 (0.01–0.44) Yes: 0.33 (0.02–6.76)	0.049 *	No: n = 24; Yes: n = 37	No: 0.005 (0.003–0.007) Yes: 0.007 (0.003–0.13)	0.061
Lung	No: n = 41; Yes: n = 14	No: 0.05 (0.07–0.77) Yes: 0.96 (0.04–5.61)	0.113	No: n = 43; Yes: n = 18	No: 0.005 (0.002–0.009) Yes: 0.008 (0.006–0.017)	0.025 *
Loco-regional	No: n = 45; Yes: n = 10	No: 0.16 (0.01–5.26) Yes: 0.24 (0.02–1.00)	0.785	No: n = 49; Yes: n = 12	No: 0.007 (0.003–0.011) Yes: 0.007 (0.003–0.007)	0.315
Lymph nodes	No: n = 43; Yes: n = 12	No: 0.16 (0.01–1.00) Yes: 0.23 (0.01–6.11)	0.514	No: n = 50; Yes: n = 11	No: 0.006 (0.002–0.009) Yes: 0.009 (0.004–0.014)	0.100
Peritoneum	No: n = 43; Yes: n = 12	No: 0.32 (0.01–5.26) Yes: 0.05 (0.01–0.15)	0.139	No: n = 50; Yes: n = 11	No: 0.007 (0.003–0.009) Yes: 0.007 (0.002–0.056)	0.799
Bone	No: n = 53 Yes: n = 2	No: 0.16 (0.01–4.69) Yes: 0.09 (0.01–0.17)	-	No: n = 59 Yes: n = 2	No: 0.007 (0.003–0.010) Yes: 0.04 (0.004–0.004)	-
**Surgery on primary site**						
Yes	n = 42	0.06 (0.01–0.92)	0.010 *	n = 47	0.008 (0.006–0.010)	0.126
No	n = 13	5.46 (0.07–9.86)		n = 14	0.006 (0.002–0.010)	
**Chemotherapy**						
No	n = 1	0.01	-	n = 1	0.007	-
Yes (any)	n = 42	0.17 (0.01–5.26)		n = 47	0.007 (0.003–0.010)	
Only synthetic agents	n = 14	0.36 (0.01–8.16)	0.359 **	n = 17	0.007 (0.005–0.007)	0.343 **
Only targeted biologics	n = 4	0.02 (0.01–0.12)		n = 5	0.013 (0.011–0.469)	
Combination of synthetic and biologics	n = 24	0.17 (0.01–3.13)		n = 25	0.005 (0.002–0.10)	
Missing	n = 12	0.25 (0.04–3.90)		n = 13	0.006 (0.002–0.009)	
**Response**						
Complete response	n = 3	0.01 (0.01–0.02)	0.160	n = 3	0.011 (0.004–0.469)	0.552
Partial response	n = 4	0.03 (0.00–3.58)		n = 4	0.006 (0.004–0.007)	
Stable disease	n = 6	0.09 (0.01–0.23)		n = 8	0.006 (0.003–0.012)	
Progressive disease	n = 17	0.44 (0.03–1.00)		n = 20	0.007 (0.003–0.010)	
TE	n = 7	0.33 (0.16–3.10)				
Missing	n = 18	0.11 (0.01–5.46)		n = 18	0.005 (0.002–0.008)	
**Survival**						
Survived	n = 22	0.11 (0.01–0.39)	0.459	n = 25	0.004 (0.002–0.007)	0.182
Deceased	n = 9	0.22 (0.01–8.16)		n = 11	0.007 (0.003–0.009)	
Missing	n = 24	0.53 (0.02–6.18)		n = 25	0.008 (0.004–0.017)	

§ excluding missing values; * statistically significant for *p* < 0.05. ** *p*-values are referred to the comparison between the three chemotherapy approaches (only synthetic agents, only targeted biologics, and their combination).

**Table 5:** MAF values of *KRAS* mutational status at the baseline and during treatment, type of therapy, and response in mCRC patients enrolled in the study. M: mutated; WT: wild type; END: end of the study; PR: partial response; PD: progressed disease; CR: complete response; SD: stable disease.

	Baseline	4 Weeks	8 Weeks	12 Weeks	48 Weeks	Therapy	Best Response
**Oncobio001**	M (0.042)	Low DNA	M (0.340)	M (0.418 + 0.156 Cd61)	**END**	FOLFIRI + BEVACIZUMAB	PD
**Oncobio002**	M (0.173)	WT (0.010)	M (0.127)			FOLFIRI + BEVACIZUMAB	SD
**Oncobio003**	M (0.012)	M (0.254)	Low DNA			FOLFIRI	CR
**Oncobio004**	M (7.103)	Low DNA	M (0.604)			XELOX	PR
**Oncobio005**	M (0.060)	M (0.530)	M (0.082)			CAPOX + BEVACIZUMAB	PR
**Oncobio006**	Low plasma vol	Low DNA				XELOX	SD
**Oncobio007**	WT (0.005)	WT (0.006)	M (0.102)			FOLFOX + VECTIBIX	PR
**Oncobio008**	WT (0.010)	M (0.105)	Low plasma vol		0.011	FOLFIRI + VECTIBIX	SD
**Oncobio009**	WT (0.005)	WT (0.010)	M (0.503)			FOLFIRI + VECTIBIX	SD
**Oncobio010**	M (0.012)	M (0.040)	**END**			CAPOX + BEVACIZUMAB	PD
**Oncobio011**	M (0.233)	M (0.038)	M (0.451)			CAPECITABINE + PANITUMUMAB	SD
**Oncobio012**	M (0.771)	**END**				CAPOX	PD
**Oncobio013**	M (8.159)	M (0.382)	**END**			FOLFOXIRI	PD
**Oncobio014**	Low plasma vol	Low plasma vol	M (0.218)	M (1.995)		CAPOX + BEVACIZUMAB	SD
**Oncobio015**	WT (0.010)	**END**				CAPOX + BEVACIZUMAB	PD
**Oncobio016**	WT (0.005)	M (0.050)	WT (0.011)			FOLFIRI + BEVACIZUMAB	
**Oncobio017**	M (0.334)	M (0.461 + 0.065 Cd117)				FOLFOX	
**Oncobio018**	Low plasma vol	M (0.251)	M (0.290)	**END**		FOLFIRI + BEVACIZUMAB	PD
**Oncobio019**	M (0.317)	M (0.351)	M (0.242)			CAPECITABINE + BEVACIZUMAB	
**Oncobio020**	M (0.026)	M (0.045)	M (0.137)		**END**	CAPOX + BEVACIZUMAB	PD
**Oncobio021**	M (0.038 + 0.056 *NRAS* Cd12)	M (0.054)	Low plasma vol	**END**		CAPOX + BEVACIZUMAB	PD
**Oncobio022**	M (0.437)	M (0.112)	Low DNA			CAPOX + BEVACIZUMAB	SD
**Oncobio023**	M (0.393)	M (0.110)	M (0.017)			FOLFOX	PR
**Oncobio024**	WT (0.003)	M (0.018)	WT (0.008)			OXALIPLATIN	PR
**Oncobio025**	M (0.162)	M (0.063)				DEGRAMONT + BEVA	
**Oncobio026**	Low plasma vol	Low plasma vol	Low plasma vol			CAPECITABINE + BEVACIZUMAB	
**Oncobio027**	M (1.004)	Low plasma vol	M (1.044)	**END**		FOLFOX + BEVACIZUMAB	PD
**Oncobio028**	Low plasma vol	M (0.015)	M (0.038)			FOLFOX	
**Oncobio029**	M (0.218)	WT (0.005)	**END**			PEMBROLIZUMAB	PD
**Oncobio030**	M (3.102)	M (0.358 + 0.047 *NRAS* Cd12)					
**Oncobio031**	M (0.021)	M (0.012)	WT (0.008)			FOLFIRI + BEVACIZUMAB	

## Data Availability

Data are available upon request.

## Supplementary Materials

The following supporting information can be downloaded at: https://www.mdpi.com/article/10.3390/cells12111458/s1, Supplementary Table S1a,b: Tissue and plasma *KRAS*, according to survival and response; Supplementary Table S2a,b: Tissue and plasma *NRAS*, according to survival and response.
