# Supplementary material for: Evaluation of RAS Mutational Status in Liquid Biopsy to Monitor Disease Progression in Metastatic Colorectal Cancer Patients
Source: Cells. 2023 May 24;12(11):1458. doi: 10.3390/cells12111458 (PMC10253213; doi:10.3390/cells12111458)
Supplement: Supplementary file 1 [file cells-12-01458-s001.zip › cells-2352765-supplementary.pdf]

SUPPLEMENTARY MATERIAL

Evaluation of *RAS* mutational status in liquid biopsy to monitor disease progression in metastatic colorectal cancer patients

Supplementary Table S1a,b: Tissue and plasma KRAS, according to survival and response

a)

	Tissue KRAS		
N obs=36 (with available survival data and tissue KRAS data)	WT	Mutated	p-value
**Survived**	13 (86.7%)	12 (57.1%)	0.077
**Deceased**	2 (13.3%)	9 (42.9%)	

	Plasma KRAS		
N obs=32 (with available survival data and plasma KRAS data)	WT	Mutated	p-value
**Survived**	5 (71.4%)	18 (72.0%)	1.000
**Deceased**	2 (28.6%)	7 (28.0%)	

b)

	Tissue KRAS			Plasma KRAS		
Response	WT (n=17)	Mutated (n=26)	p-value	WT (n=9)	Mutated (n=29)	p-value
Complete response	1 (5.9%)	2 (7.7%)	0.940	1 (11.1%)	2 (6.9%)	0.163
Partial response	2 (11.8%)	2 (7.7%)		2 (22.2%)	2 (6.9%)	
Stable disease	4 (23.5%)	4 (15.4%)		3 (33.3%)	4 (13.8%)	
Progressive disease	7 (41.2%)	13 (50.0%)		3 (33.3%)	14 (48.3%)	
TE	3 (17.7%)	5 (19.2%)		0	7 (24.1%)	

Supplementary Table S2a,b: Tissue and plasma NRAS, according to survival and response

a)

	Tissue NRAS		
N obs=36 (with available survival data and tissue KRAS data)	**WT**	**Mutated**	**p-value**
**Survived**<br>**Deceased**	25 (69.4%)<br>11 (30.6%)	0<br>0	-
	**Plasma NRAS**		
N obs=36 (with available survival data and plasma NRAS data)	**WT**	**Mutated**	**p-value**
**Survived**<br>**Deceased**	24 (70.6%)<br>10 (29.4%)	1 (50.0%)<br>1 (50.0%)	0.524

b)

	Tissue NRAS	Plasma NRAS		
**Response**	**WT (n=43)**	**WT (n=39)**	**Mutated (n=4)**	**p-value**
Complete response	3 (7.0%)	2 (5.1%)	1 (25.0%)	0.274
Partial response	4 (9.3%)	4 (10.3%)	0	
Stable disease	8 (18.6%)	8 (20.5%)	0	
Progressive disease	20 (46.5%)	17 (43.6%)	3 (75.0%)	
TE	8 (18.6%)	8 (20.5%)	0	
